# Sp1 Targeted PARP1 Inhibition Protects Cardiomyocytes From Myocardial Ischemia–Reperfusion Injury via Downregulation of Autophagy

**DOI:** 10.3389/fcell.2021.621906

**Published:** 2021-05-25

**Authors:** Yifeng Xu, Boqian Wang, Xiaoxiao Liu, Yunfei Deng, Yanqi Zhu, Feng Zhu, Yanyan Liang, Hongli Li

**Affiliations:** ^1^Department of Cardiology, Shanghai General Hospital, School of Medicine, Shanghai Jiao Tong University, Shanghai, China; ^2^State Key Laboratory of Oncogenes and Related Genes, Institute for Personalized Medicine, School of Biomedical Engineering, Shanghai Jiao Tong University, Shanghai, China

**Keywords:** myocardial ischemia-reperfusion injury, oxygen-glucose deprivation/reperfusion, PARP1, Sp1, autophagy

## Abstract

Myocardial ischemia–reperfusion injury (MIRI), characterized by post-ischemic cardiomyocytes death and reperfusion myocardial damage, is a lethal yet unresolved complication in the treatment of acute myocardial infarction (AMI). Previous studies have demonstrated that poly(ADP-ribose) polymerase-1 (PARP1) participates in the progression of various cardiovascular diseases, and various reports have proved that PARP1 can be a therapeutic target in these diseases, but whether it plays a role in MIRI is still unknown. Therefore, in this study, we aimed to explore the role and mechanism of PARP1 in the development of MIRI. Firstly, we demonstrated that PARP1 was activated during MIRI-induced myocardial autophagy *in vitro*. Moreover, PARP1 inhibition protected cardiomyocytes from MIRI through the inhibition of autophagy. Next, we discovered that specificity protein1 (Sp1), as a transcription factor of PARP1, regulates its target gene PARP1 through binding to its target gene promoter during transcription. Furthermore, silencing Sp1 protected cardiomyocytes from MIRI via the inhibition of PARP1. Finally, the functions and mechanisms of PARP1 in the development of MIRI were also verified *in vivo* with SD rats model. Based on these findings, we concluded that PARP1 inhibition protects cardiomyocytes from MIRI through the inhibition of autophagy, which is targeted by Sp1 suppression. Therefore, the utilization of PARP1 exhibits great therapeutic potential for MIRI treatment in future.

## Introduction

Acute myocardial infarction (AMI), associated with high mortality and morbidity, is one of the major causes of death in the world (Penna et al., 2013; Pu et al., 2014). With the application of reperfusion therapies, such as percutaneous coronary intervention (PCI) and thrombolysis, that restore the blood flow to the ischemic area, the impaired myocardium can be salvaged (Xuan and Jian, 2016; González-Montero et al., 2018). However, myocardial reperfusion is likely to cause an excessive production of reactive oxygen species (ROS), contributing to the activation of autophagy, the induction of post-ischemic cardiomyocyte death, and the generation of cardiac dysfunction, which was termed as myocardial ischemia-reperfusion injury (MIRI) (Movassagh and Foo, 2008; Gustafsson and Gottlieb, 2009; Morales et al., 2014). Accompanied by clinical complications like reperfusion arrhythmias, lethal reperfusion, myocardial stunning and no-reflow (González-Montero et al., 2018), MIRI is a common yet fatal clinical disease which remains to be solved. Therefore, it is of urgent demand to explore the molecular mechanisms of MIRI and to develop novel molecular interventions that may reduce the occurrence of MIRI and improve the prognosis of AMI patients (He et al., 2014).

Poly(ADP-ribose) polymerase-1 (PARP1), characterized by attaching ADP-ribose polymer chains to the target proteins and promoting DNA repair, is a post-translational modification enzyme (Kim et al., 2004, 2005; Pirrotta, 2004). Previous studies have shown that PARP1 is associated with many cardiovascular diseases, including hypertension, atherosclerosis, myocardial hypertrophy and circulatory shockn (Molnár et al., 2006; Pacher and Szabó, 2007; Esposito and Cuzzocrea, 2009; Xu et al., 2014). It has been demonstrated that PARP1 can be activated by starvation (Wang H. et al., 2018), angiotensin II (Wang et al., 2013) and high glucose (Jagtap and Szabo, 2005). Additionally, evidence showed that PARP1 overexpression can bring about damage to cardiac structure and cardiac function during the progression of heart failure (Pillai et al., 2005), whereas PARP1 inhibition can prevent cardiac remodeling (Pillai et al., 2006), apoptosis, fibrosis and inflammation (Gilad et al., 1997; Wayman et al., 2001; Jagtap and Szabo, 2005; Wang et al., 2013, Wang H. et al., 2018). Therefore, PARP1 inhibition was proved to be cardiac-protective in heart failure and post-infarction myocardial remodeling (Halmosi et al., 2016), but whether it is involved in MIRI is unknown.

Specificity protein 1(Sp1) transcription factor (TF) is a member of the Sp/Kruppel-like factor family, which is involved in embryonic development and cell cycle regulation (Safe et al., 2014). Generally, the biological function of Sp1 is activated when it is binding to its target DNA binding sites (Chintharlapalli et al., 2007). Furthermore, it was reported that down-regulation of Sp1 and Sp1-related genes are considered as drug-dependent (Abdelrahim et al., 2007). It has been reported that Sp1 is closely related to cancers, and high level of Sp1 in human blood is perceived as a negative prognostic factor to cancers (Mertens-Talcott et al., 2007; Chadalapaka et al., 2008, 2013; Chintharlapalli et al., 2009, 2011; Papineni et al., 2009; Colon et al., 2011). However, the role of Sp1 in the progression of MIRI remains unclear.

Autophagy, also nominated as type II programmed cell death, plays an essential role in the pathophysiological progression of MIRI (Thapalia et al., 2014). It is a process of transporting damaged, denatured or aged organelles to lysosomes for degradation, and the formation of double-membraned autophagosomes are the symbol of autophagy, during which the activation of Beclin1, LC3 (Micro-tubule-associated protein1 light chain 3) and ATG12 are characteristic biomarkers to trigger this process (Ma et al., 2012; Zou et al., 2019). Under physiological conditions, autophagy maintains intracellular homeostasis, and thus it assists in preserving normal function and survival of cells; while under pathological circumstances, autophagy leads to a variety of diseases, such as inflammation (Virgin and Levine, 2009), aging (Rubinsztein et al., 2011), cancer (Yang et al., 2011), metabolic syndrome (Codogno and Meijer, 2010), liver disease (Hidvegi et al., 2010) and heart disease (Nakai et al., 2007). When it comes to ischemic heart diseases, autophagy is beneficial and adaptive during ischemia, but harmful and fatal during reperfusion. As expected, MIRI is the result of this autophagy, which is out of control. In the previous ischemic area, the existence of coronary microvascular occlusion sustains for 48 h, while the autophagy effect lasts for 72 h. Due to the intracellular autophagy, the final clinical manifestation of MIRI is presented as expanded infarct myocardium size, deteriorated left ventricular ejection fraction, aggravated left ventricular remodeling and worse clinical prognosis (Thapalia et al., 2014). Therefore, the regulation of autophagy is an potential target for the intervention of MIRI.

In this study, we have discovered the role and mechanism of PARP1 in the progression of MIRI. Function experiments indicated that PARP1 was activated by MIRI-induced myocardial autophagy. By *in vivo* and *in vitro* experiments, we demonstrated that inhibiting PARP1 could alleviate myocardial injury triggered by MIRI whereas promoting autophagy could reverse the myocardial protection effect of PARP1 inhibition. Further exploration of the mechanism identified that PARP1 inhibition protects cardiomyocytes from MIRI through inhibition of autophagy, which is targeted by Sp1 suppression.

## Materials and Methods

### Cell Culture and Establishment of Oxygen-Glucose Deprivation/Reperfusion (OGD/R) Model

A cellular model of Oxygen-Glucose Deprivation/Reperfusion (OGD/R) was adopted to simulate *in vivo* myocardial ischemia reperfusion. Briefly, embryonic rat heart-derived H9c2 cells were cultured in 6-well plates in a humidified and 5% CO_2_ atmosphere at 37°C, with DMEM supplemented with 10% FBS. When the cells grew to about 80% density, the medium of the OGD/R group was replaced with sugar-free medium that had been prefilled with 95% N_2_ and 5% CO_2_ for 30 min to replace the oxygen in the solution, and then the cells were placed in an anoxic incubator, filled with 95% N_2_ and 5% CO_2_ mixed gas. After 6 h of OGD, the cells were transferred into the regular incubator for reperfusion, placed for 2 h, and then collected for subsequent assays (Supplementary Figure 1).

### Cell Transfection

H9c2 cells were seeded in 60 mm cell culture dishes and cultured in medium without antibiotics overnight. To knockdown the expression of Sp1, cells at 40% confluence were transfected with Sp1 shRNA, using Lipofectamine 2000 reagent according to the manufacturer’s instructions. At 36 h after transfection, H9c2 cells were incubated in ischemic conditions for 12 h, as previously described.

### Real-Time PCR

Total RNA was isolated from H9c2 cells with TRIzol Reagent (Ambion, 15596026, Texas, United States). PrimeScriptTM RT reagent Kit (TAKARA, RR037A, Takara Bio, Otsu, Japan) was adopted to determine the mRNA expression level. According to the manufacturer’s instructions, real-time PCR was performed with related forward and reverse primers with SYBR^®^ Premix Ex Taq^TM^ II (Takara, RR420A, Takara Bio, Otsu, Japan) in real-time system (Roche, LightCycler^®^ 96, Switzerland). The relative mRNA expression levels were calculated using 2^–ΔΔCt^ method and were presented as fold changes relatively to the expression levels of the control group. The following primers were used in the real-time PCR: GAPDH forward, 5′-CCTGCACCACCAACTGCTTA-3′ and reverse, 5′-CATCAC GCCACAGCTTTCCA-3′; PARP1 forward, 5′-ACCACGCACA ATGCCTATGA-3′ and reverse, 5′-AGTCTCCGGTTGTGAAG CTG-3′; Beclin-1 forward, 5′-AGCCTCTGAAACTGGACACG-3′ and reverse, 5′-CCTCTTCCTCCTGGCTCTCT-3′; LC3 forward, 5′-CCGTAGTTCGCTGTACGAGG-3′ and reverse, 5′-CCGTAGTTCGCTGTACGAGG-3′; ATG-12 forward, 5′-GCTG AAGGCTGTAGGAGACAC-3′ and reverse, 5′-GGAAGGGGCA AAGGACTGATT-3′; Gabarapl-1 forward, 5′-AGAGGACCAC CCCTTCGAATA-3′ and reverse, 5′-GAGCCTTCTCCACGATG ACC-3′.

### Western Blot Analysis

Total protein was extracted from H9c2 cells with RIPA lysis buffer (dilution:1:1000, Beyotime, P0013B, Nan Tong, China) supplemented with PMSF (Amresco, 329-98-6, United States). The protein lysates were resolved with SDS-PAGE gels and electro-transferred onto a nitrocellulose membrane (Millipore, HATF00010, Germany). At room temperature, TBST buffer with 5% non-fat milk powder (Sangon Biotech, NB0669-250g, Shanghai, China) was used to block the membranes for 1.5 h. At 4°C, related primary antibodies were incubated with the membrane overnight. At room temperature, the membrane was washed with PBS (Genom, GNM20012, Hangzhou, China) and incubated with a horseradish peroxidase labeled secondary antibody (Beyotime, A0208, A0216, Nantong China) for 1 h. The membrane was analyzed with the Chemiluminescence imaging system (Clinx, ChemiQ4600, Shanghai, China). The relative protein expression levels were presented as fold changes relative to the expression levels of the control group. Antibodies against Cleaved PARP1 (Abcam, ab32064, 1:1000, Cambridge, United Kingdom), Beclin1 (Abcam, ab207612, 1:2000, Cambridge, United Kingdom), Gabarapl1 (Abcam, ab86497, 1:1000, Cambridge, United Kingdom), ATG12 (Abcam, ab155589, 1:1000, Cambridge, United Kingdom), LC3A/B (Cell Signaling Technology CST, 12741, 1:1000, MA, United States), GATA1 (Abbexa, abx121608, 1:10, United Kingdom), Sp1(Abcam, ab13370, 1:10, Cambridge, United Kingdom), YYI (Abcam, ab109237, 1:10, Cambridge, United Kingdom), CTCF (Abcam, ab70303, 1:10, Cambridge, United Kingdom), β-actin (Abcam, ab8227, 1:5000, Cambridge, United Kingdom), GAPDH (Abcam, ab181602, 1:10000, Cambridge, United Kingdom) were used as primary antibodies. Cleaved PARP1 is an active form of PARP1 and was used in this study to represent the expression of PARP1.

### Immunofluorescence Microscopy

The H9c2 cells were seeded onto sterilized coverslips, and cultured in the 5% CO_2_ incubator (Corning, Wuxi, China) at 37 °C for about 6 h. The cells were then rinsed with PBS, fixed with 4% paraformaldehyde (Sinopharm, Shanghai, China) for 60 min, treated with 0.5% TritonX-100 for 20 min, and blocked with 3% H_2_O_2_ for 15 min, all at room temperature. Afterward, related primary antibodies were incubated with the cells overnight at 4°C. At room temperature, the cells were washed with PBS and incubated with fluorescent secondary antibodies for 2 h, followed by staining with DAPI (10 μg/ml, Beyotime, Nantong, China) for 5 min. Cells were observed and imaged with a fluorescence microscope (Leica, Germany).

### Flow Cytometry

The H9c2 cell culture medium was sucked out into a suitable centrifuge tube. Then the adherent H9c2 cells were washed by PBS once and digested by trypsin cell digestion solution, which contained EDTA (Solarbio, T1300-100, Beijing, China). After incubation at room temperature, we added the cell culture medium which was collected before, and transfered it to the centrifugal tube, then centrifuged 1000 *g* for 5 min (Bioridge, TDZ4-WS, Shanghai, China). The H9c2 cells were centrifuged and resuspended with 195 μl Annexin V-FITC binding solution and 5 μl Annexin V-FITC (Beyotime, C1062, Nantong, China), incubated at 4°C for 15 min. Then, the cells were centrifuged again and resuspended with 190 μl Annexin V-FITC binding solution and 10 μl propidium iodide staining solution (Beyotime, Nantong, China), incubated at 4°C for 5 min. The fluorescence was detected by flow cytometer (BD, Accuri C6, United States).

### MTT Assay

The H9c2 cells were cultured with 96-well plates. After the treatments of each experimental group, the culture medium was replaced with 20 μl MTT solution (Beyotime, Nantong, China). After incubation at 37°C for 4 h, the supernatant in each well was removed, replaced with 150 μl DMSO (Sigma, Germany). The plate was shaken for 10 min at a low speed to fully dissolve the crystals. The absorbance was measured at 490 nm by plate reader (Thermo, Carlsbad, CA, United States).

### Chromatin Immunoprecipitation (ChIP)

Proteins and DNA of proliferating H9c2 cells were crosslinked at room temperature for 10 min with 10 ml Fix Buffer (10 mM HEPES pH = 7.6, 1 M sucrose, 5 mM KCl, 5 mM MgCl_2_, 1% formaldehyde, 14 mM mercaptoethanol, 0.6% TritonX-100, and 0.4 mM PMSF, all from Sangon Biotech and Sinopharm). To terminate the crosslinking, 0.8 ml 2 M Glycine (Sigma, Germany) was added. The cells were placed on ice for 30 min before being scraped down and transferred to a pre-cooled centrifugal tube, centrifuged for 20 min at 4 000 rpm, 4°C. The supernatant was removed and cells were resuspended with 1 mL pre-cooled Extraction Buffer (0.4 M sucrose, 10 mM MgCl_2_, 5 mM mercaptoethanol, 10 nM Tris-HCl pH = 8.0, and 1% proteinase inhibitor; Tris-HCl and proteinase inhibitor were from Sigma, Germany). The sample was crushed by ultrasonic (Sonics, United States). The pyrolysis solution was diluted by 10 times with pre-cooled Chip Buffer (1.1%TritonX-100, 1.2 mM EDTA, 16.7 mM Tris-HCl pH = 8.0, and 167 mM NaCl from Sinopharm, Shanghai, China). Protein A agarose beads (Cell Signaling Technology, MA, United States) were prepared with salmon sperm DNA (Sigma, Germany), and rinsed for three times with Chip Buffer. Then the pyrolysis solution was mixed with Chip Buffer, centrifuged at 4°C, 13000 rpm for 30 s. The pyrolysis solution was preserved at –20°C for Input. TE Buffer (10 mM Tris-HCl, 1 mM EDTA pH = 8.0), NaCl, SDS (Sinopharm, Shanghai, China) were added to Input. Protein K (TIANGEN, Beijing, China) was added to immune complex. The supernatant was extracted by adding chloroform (Sangon Biotech, Shanghai, China) of equal volume at room temperature and centrifuging at 13000 rpm for 15 min. DNA samples were added to the centrifugal tube overnight at −20°C with NaAc (Aladdin, Shanghai, China), ethanol (Sangon Biotech, Shanghai, China) and glycogen (Sinopharm, Shanghai, China). The DNA sample was washed with 70% ethanol, dried in incubator at 37 °C and precipitated with 50 μl 10 mM Tris-HCl (pH = 7.5), before its concentration was determined and adjusted for the subsequent qRT-PCR experiment.

### Dual-Luciferase Reporter Assay

Due to the high transfection efficiency of 293T cells, 293T cells were used and incubated on 24-well plates at 37°C. When the cells grew to about 70–80% density, the medium was replaced with serum-free MEM (Gibco, Carlsbad, CA, United States) without antibiotics, incubated overnight. The plasmid and Lipofectamine^TM^ 2000 (Invitrogen, Carlsbad, CA, United States) were, respectively, diluted with MEM, and incubated at room temperature for 5 min. Then they were fully mixed and placed at room temperature for 20 min. The medium in the 24-well plate was replaced with the mixture. After 4–6 h of incubation, the transfection solution was replaced with MEM containing 10% FBS. After 48 h of transfection, the cells were fully lysed. Bright-Lumi^TM^ II Firefly Luciferase Reporter Gene Assay Kit and Renilla-Lumi^TM^ Luciferase Reporter Gene Assay Kit (Beyotime, Nantong, China) and the fluorescein enzyme detection buffer of sea kidney (Beyotime, Nantong, China) were used to detect the luciferase activity with a plate reader (Promega, United States). The RLU (relative light unit) was determined by mixing 100 μl samples with 100 μl reagent solution. Cell lysate without regent was used as blank control.

### Establishment of the Rat MIRI Model

The study was conducted on 42 male SD rats (2 ± 1 months of age, weighing 220 ± 20g) bought from Shanghai SLAC Laboratory Animal Co., Ltd (project number SCXK 2017-0005). The rats were anesthetized with isoflurane. A four-limb electrocardiogram (ECG) was recorded to monitor ST segment amplitude changes. After removing the hair on the chest of rats, the thorax was opened, and the heart was exposed via left thoracotomy in the fourth intercostal space. A 6–0 silk ligature (Solarbio, Beijing, China) was used to ligate the left anterior descending coronary artery (LAD). Ischemia was monitored and confirmed visually via the ST segment elevation on ECG and prompt and sustained pallor of the anterior wall distal to the ligation site. After 45 min of ischemia, the ligature was loosened for 24 h, and reperfusion was confirmed by prompt return of color to the myocardium. Sham control group rats were treated with the same surgical procedures except the ligation of the left coronary artery. The AD vector packed with PARP1. The Control group and the I/R group were administered with the intragastrical injection of Sp1 shRNA NC. In contrast, the I/R + Sp1 shRNA and I/R+ AD-PARP1 group were given a 30 μl injection of recombinant Sp1 shRNA and AD-PARP1 around the infarction region, respectively.

The rats were randomly divided into seven groups (n = 5 for each groups). The groups were assigned as follows: (1) sham (0.9% normal saline, Sham group), (2) I/R (0.9% normal saline, I/R group), (3) I/R + AG-14361 at a dose of 5 mg/kg (Intraperitoneal injection once a day for 5 days), (4) I/R +AG-14361+BEZ235 at a dose of 50 mg/kg (Intraperitoneal injection), (5) I/R +SP1 shRNA NC (10^8^ CFU/100 g weight), (6) I/R +SP1 shRNA (10^8^ CFU/100 g weight), (7) I/R + AD-PARP1 (10^8^ CFU/100 g weight).

### Masson’s Trichrome Staining

Masson’s trichrome staining was done with Masson’s Trichrome Stain Kit (Shanghai Xinfan Biotechnology Co., Ltd, Shanghai, China), according to the manufacturer’s instructions. The Sections were fixed in 4% formaldehyde and embedded in paraffin. The stains of sections were observed and imaged with optical microscopy (Olympus, Japan).

### Hematoxylin and Eosin (H&E) Staining

The myocardial tissues were fixed, dehydrated, transparent, soaked in wax, embedded in paraffin and sectioned. Then they were stained with hematoxylin and eosin (Sinopharm, Shanghai, China), clarified with xylene (Beijing Leagene Co., Ltd, Beijing, China) and sealed with neutral balsam (Sinopharm, Shanghai, China). The sections were observed and imaged with optical microscopy (Olympus, Japan).

### TUNEL Assay

According to the manufacturer’s instructions (KeyGEN BioTECH, Nanjing, China), cells were fixed in 4% paraformaldehyde solution for 30 min at room temperature, and counterstained with DAPI (10 μg/ml, Beyotime, Nantong, China). The slides were sealed and visualized by fluorescence microscopy (Leica, Germany).

### Statistical Analysis

All data were presented as means ± standard deviation, and all the experiments were performed at least three times. Statistics were done with SPSS version 22.0. The one way-ANOVA test was used for comparison between multiple groups, while multiple intra-groups comparisons were achieved through SNK test. When *P*-value is below 0.05, the change is considered statistically significant.

## Results

### PARP1 Was Activated by OGD/R Induced Myocardial Autophagy

**FIGURE 1:**
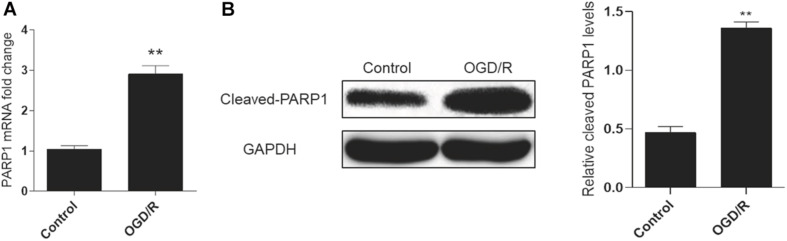
PARP1 was activated by OGD/R-induced myocardial autophagy. H9c2 cells were cultured in an oxygen/glucose-deprived incubator for 6 h, and then reperfused by oxygen and glucose for 2 h to cultivate an OGD/R model. **(A)** Real-time PCR showed the mRNA level of PARP1 increased when H9c2 cells were exposed to OGD/R. **(B)** Western blot showed the protein level of PARP1 also rose when H9c2 cells were exposed to OGD/R. Compared with control group, **P < 0.01. *N* = 4 for each group.

A cell model of oxygen-glucose deprivation/reperfusion (OGD/R) was adopted to simulate MIRI *in vitro* and induce autophagy. H9c2 cells were cultured in an oxygen-glucose-deprived incubator for 6 h, and then reperfused by oxygen and glucose for 2 h to cultivate the OGD/R model. Real-time PCR analysis showed that the mRNA expression of *PARP1* increased in the OGD/R model, compared to control group (Figure 1A). Meanwhile, the result of western blot also showed the expression of PARP1 was increased, compared to that in control group (Figure 1B).

### PARP1 Inhibition Protected Cardiomyocytes From OGD/R Through Inhibition of Autophagy

**FIGURE 2:**
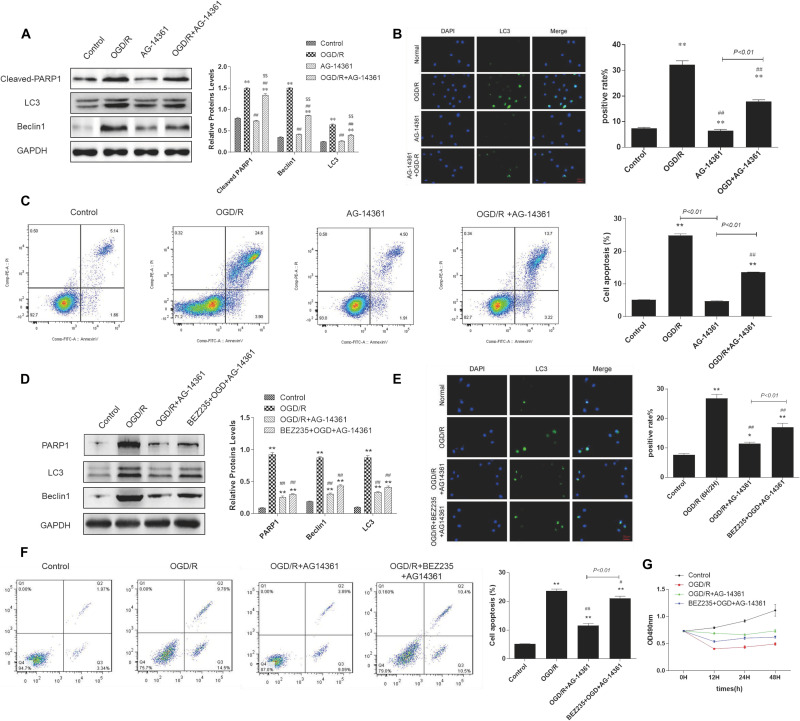
PARP1 inhibition protected cardiomyocytes from OGD/R through inhibition of autophagy. The OGD/R-modeled H9c2 cells were treated with PARP1 inhibitor AG-14361 (10 μM) for 1 h and a novel autophagy promoter NVP-BEZ235 (0.25 μM) for 24 h. **(A)** Western blot showed the expression of PARP1 and autophagy related proteins rose when H9c2 cells were treated with AG-1436. **(B)** GFP-LC3 assay showed the different level of GFP puncta between control group and other groups. Magnification: 630×. **(C)** Flow cytometry analysis assessed the apoptotic rate of control group and other groups. **(D)** Western blot assessed the expression of PARP1 and autophagy related proteins when H9c2 cells was treated with PARP1 inhibitor and BEZ235. **(E)** GFP-LC3 assay showed the different level of GFP puncta between control group and other groups. Magnification: 630×. **(F)** Flow cytometry analysis showed the apoptotic rate of control group and other groups. **(G)** MTT assay showed the OD value of cells in control group and other groups. Compared with control group, *P < 0.05, **P < 0.01; compared with OGD/R group, ^#^*P* < 0.05, ^##^*P* < 0.01; Compared with AG-14361 group, ^$^*P* < 0.05, ^$$^*P* < 0.01. *N* = 4 for each group.

To examine whether PARP1 inhibition could protect cardiomyocytes from MIRI, we used PARP1 inhibitor AG-14361 (S2178, Selleck Chemicals) to suppress the expression of PARP1 (Smith et al., 2016). After treating H9c2 cells with OGD/R model, western blot showed that compared to control group, the expression of PARP1 and autophagy related proteins such as LC3 and Beclin1 increased, indicating that the autophagy related cardiac injury was induced by OGD/R. However, treating cardiomyocytes with 10 μM AG-14361 for 1 h (Vazquez-Ortiz et al., 2015; Deng and Mi, 2016), we observed that the expression of PARP1 and autophagy related proteins were downregulated (Figure 2A). Simultaneously, we observed that in the GFP-LC3 assay, compared to control group, the number of GFP puncta increased in H9c2 cells when exposed to OGD/R, but reduced when treating cardiomyocytes with AG-14361 (Figure 2B). It was also detected by flow cytometry that the number of necrotic and apoptotic H9c2 cells increased when exposed to OGD/R. Treating cardiomyocytes with AG-14361, we discovered that the percentage of apoptotic cells decreased (Figure 2C). Therefore, PARP1 inhibition protected cardiomyocytes from OGD/R.

To further study the way that PARP1 inhibition protect cardiomyocytes from MIRI, we use NVP-BEZ235 (Selleck Chemicals), a novel autophagy promoter, to increase the effect of autophagy related cardiac injury (Liu et al., 2018). After treating H9c2 cells with 0.25 μM BEZ235 for 24 h, western blot analysis indicated that the expression of PARP1 and autophagy-related proteins (LC3 and Beclin1) increased again, compared to the group in which cardiomyocytes were treated with AG-14361 (Figure 2D). Identically, GFP-LC3 assay, which assessed autophagic flux, confirmed the same result. Compared to control group, the number of GFP puncta increased in H9c2 cells when exposed to OGD/R, indicating that autophagic flux was upregulated under OGD/R treatment. Treating cardiomyocytes with AG-14361, we observed that GFP puncta reduced, whereas treating cardiomyocytes with BEZ235, GFP puncta increased again (Figure 2E). At the same time, flow cytometry analysis revealed that the number of necrotic and apoptotic H9c2 cells increased when exposed to OGD/R, compared to control group. Treating cardiomyocytes with AG-14361, we detected that the percentage of necrotic and apoptotic cells reduced, whereas treating cardiomyocytes with BEZ235, the percentage of necrotic and apoptotic cells was increased again (Figure 2F). Accordingly, as showed in MTT assay, the cell viability of H9c2 cells decreased when exposed to OGD/R, compared to control group. Treating cardiomyocytes with AG-14361, we identified that the cell viability of H9c2 cells increased, whereas treated with BEZ235, the cell viability of H9c2 cells decreased again (Figure 2G). As is mentioned above, treating cardiomyocytes with BEZ235 could reverse the myocardial protection effect of PARP1 inhibition.

Taken together, these results demonstrated that PARP1 inhibition can protect cardiomyocytes from OGD/R through inhibition of autophagy.

### Sp1 Is a Transcription Factor of PARP1 That Regulates Its Expression During Transcription

**FIGURE 3:**
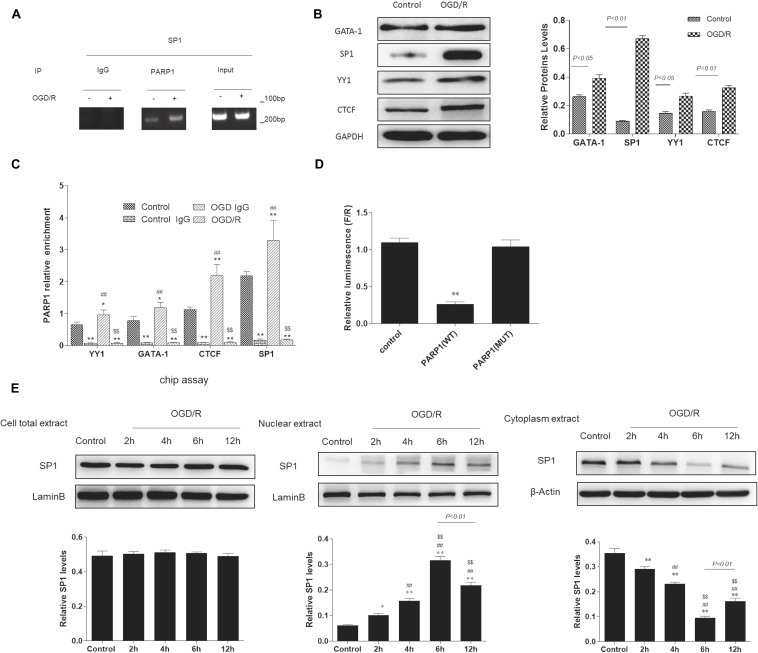
Sp1 is a transcription factor of PARP1 that regulates the target gene of PARP1 during transcription. **(A)** In ChIP assays, the DNA fragment size of target protein antibody PARP1 and input group IP were 100-200 bp. IgG was served as a negative control and input was a positive control. **(B)** Western blot analysis showed the expression of different transcription factors. Compared with control group, *P < 0.05, **P < 0.01. **(C)** Real-time PCR showed the mRNA expressions of PARP1 which were recruited by different transcription factors. Compared with control group, *P < 0.05, **P < 0.01; compared with control IgG group, ^##^*P* < 0.01; compared with OGD/R group, ^$$^*P* < 0.01. **(D)** The fluorescein value in the dual-luciferase reporter assay decreased significantly after mutation of PARP1, indicating that there was a targeted regulation between Sp1 and PARP1. Compared with control group, **P < 0.01. **(E)** Western blot was performed in nuclear extract level, cytoplasm extract level and cell total extract level to show different protein expression levels of Sp1. Compared with control group, *P < 0.05, **P < 0.01; compared with OGD/R-2 h group, ^#^*P* < 0.05, ^##^*P* < 0.01; compared with OGD/R-4 h group, ^$^*P* < 0.05, ^$$^*P* < 0.01. *N* = 4 for each group.

According to bioinformatics analysis^1^, *Sp1, YY1, CTCF, GATA-1* were selected as transcription factors of PARP1. To determine whether there exists a targeted regulation between Sp1 and PARP1, ChIP assay was conducted. We used IgG as negative control and input as positive control. After ChIP verification, IgG did not show any band. The DNA fragment size of target protein antibody PARP1 and input group IP were 100–200 bp, which matched with the requirements of ChIP experiment (Figure 3A). Furthermore, Western blot analysis indicated that when exposed to OGD/R model, the expression of Sp1 was the highest among these transcription factors (Figure 3B). We also performed real-time PCR to show that compared with control group, the mRNA expression of PARP1 recruited by all these transcription factors was the highest when exposed to OGD/R model (Figure 3C). To confirm the relationship between PARP1 and Sp1, dual-luciferase reporter assay was performed. The results showed that the fluorescence decreased significantly after mutation of PARP1, indicating that there was a targeted regulation between Sp1 and PARP1 (Figure 3D). To further confirm that it was during transcription that Sp1 regulate the biological effect of PARP1, western blot was performed in nuclear extract level, cytoplasm extract level and cell total extract level. The results showed that in nuclear extracts from OGD/R-exposed H9c2 cells, the expression of Sp1 significantly increased with time and reached the peak at 6 h, whereas it simultaneously decreased with time and hit the bottom at 6 h in cytoplasm extracts. At the same time, in cell total extract level, the expression of Sp1 was almost constant over time (Figure 3E). Therefore, Sp1 regulates the biological effect of PARP1 during transcription, as the transcription occurs in nuclear level (Mollion et al., 2018).

Altogether, these results supported that Sp1 is a transcription factor of PARP1, and it regulates the target gene of PARP1 during transcription.

### Silencing Sp1 Prevented Cardiomyocytes From OGD/R

Though we have confirmed that *Sp1* is a transcription factor of PARP1, whether Sp1 has similar biological effect as PARP1 in the pathophysiological progression of OGD/R-induced H9c2 cells is still unknown. Therefore, we transfected H9c2 cells with Sp1 shRNA and negative control of Sp1 shRNA-NC for 72 h to explore the role of Sp1 in OGD/R.

**FIGURE 4:**
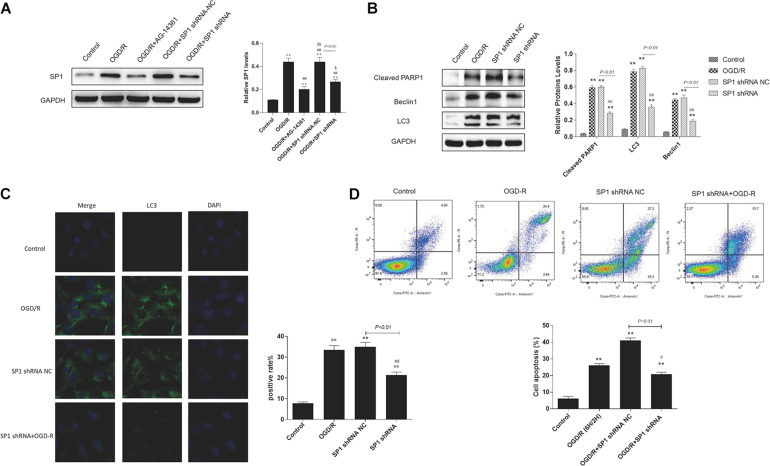
Silencing Sp1 prevented cardiomyocytes from OGD/R. The OGD/R-modeled H9c2 cells were treated with Sp1 shRNA-NC and Sp1 shRNA. **(A)** Western blot assay showed the expression level of Sp1 when transfecting H9c2 cells with Sp1 shRNA and Sp1 shRNA-NC. **(B)** Western blot showed the expression of PARP1 and autophagy related proteins when treating H9c2 cells with Sp1 shRNA and Sp1 shRNA-NC. **(C)** GFP-LC3 assay showed the number of GFP puncta in H9c2 cells when treating H9c2 cells with Sp1 shRNA and Sp1 shRNA-NC. Magnification: 630×. **(D)** Flow cytometry analysis showed the number of necrotic and apoptotic H9c2 cells when treating H9c2 cells with Sp1 shRNA and Sp1 shRNA-NC. Compared with control group, *P < 0.05, **P < 0.01; compared with OGD/R group, ^#^*P* < 0.05, ^##^*P* < 0.01. *N* = 4 for each group.

Western blot analysis showed that the expression level of Sp1 was evidently increased when exposed to OGD/R, whereas transfecting H9c2 cells with Sp1 shRNA could decrease the expression level of Sp1, compared to the Sp1 shRNA-NC-transfected cells (Figure 4A).

Furthermore, we performed western blot to demonstrated that compared to control group, the expression of PARP1 and autophagy related proteins increased when H9c2 cells were exposed to OGD/R, while treating H9c2 cells with Sp1 shRNA, we observed that the expression of PARP1 and autophagy related proteins decreased, compared to Sp1 shRNA-NC group (Figure 4B). Consistently, in GFP-LC3 assay, compared to control group, the number of GFP puncta rose in H9c2 cells when exposed to OGD/R, and declined when treating H9c2 cells with Sp1 shRNA, compared to Sp1 shRNA-NC group (Figure 4C). We also observed by flow cytometry that compared to control group, the number of necrotic and apoptotic H9c2 cells increased when exposed to OGD/R, while treating H9c2 cells with Sp1 shRNA, the number dropped compared to Sp1 shRNA-NC group (Figure 4D). Therefore, silencing Sp1 prevented cardiomyocytes from OGD/R.

### Sp1 Suppression Prevented Cardiomyocytes From OGD/R Through PARP1 Inhibition

We have demonstrated that silencing Sp1 can prevent cardiomyocytes from OGD/R, but the potential mechanism under this phenomenon remains unclear. Since we have proved that there was a targeted regulation between Sp1 and PARP1, we still transfected H9c2 cells with Sp1 shRNA as well as Sp1 shRNA-NC for 72 h and treating cardiomyocytes with 10 uM AG-14361 for 1 h to explore the association between Sp1 and PARP1 in the development of OGD/R in H9c2 cells.

**FIGURE 5:**
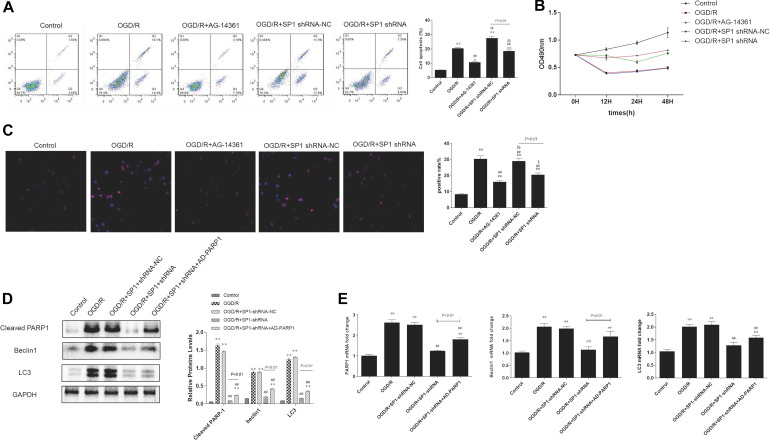
Sp1 suppression prevented cardiomyocytes from OGD/R through PARP1 inhibition. The OGD/R-modeled H9c2 cells were treated with Sp1 shRNA-NC, Sp1 shRNA, PARP1 inhibitor AG-14361 and AD-PARP1 adenovirus. **(A)** Flow cytometry analysis showed that the percentages of necrotic and apoptotic cells when treating H9c2 cells with Sp1 shRNA, Sp1 shRNA-NC and AG-14361. **(B)** MTT assay was performed to analyze the cell viability of H9c2 cells when treating H9c2 cells with Sp1 shRNA, Sp1 shRNA-NC and AG-14361. **(C)** Immunofluorescence showed the positive rate of Sp1 when treating H9c2 cells with Sp1 shRNA, Sp1 shRNA-NC and AG-14361. Magnification: 200×. **(D)** Western blot showed the expression of PARP1 and autophagy related proteins when treating H9c2 cells with Sp1 shRNA, Sp1 shRNA-NC and AD-PARP1 adenovirus. **(E)** Real-time PCR showed the mRNA level of PARP1 and autophagy related proteins when treating H9c2 cells with Sp1 shRNA, Sp1 shRNA-NC and AD-PARP1 adenovirus. Compared with control group, *P < 0.05, **P < 0.01; compared with OGD/R group, ^#^*P* < 0.05, ^##^*P* < 0.01; compared with OGD/R+AG-14361 group, ^$^*P* < 0.05, ^$$^*P* < 0.01. *N* = 4 for each group.

Flow cytometry analysis showed that the percentages of necrotic and apoptotic cells was decreased in Sp1 shRNA or AG-14361 transfected cells, whereas treating cardiomyocytes with Sp1 shRNA-NC, the percentages of necrotic and apoptotic cells was increased again (Figure 5A). In support of this, MTT assay was performed to analyze the cell viability of H9c2 cells. Results showed that the cell viability of Sp1 shRNA or AG-14361 transfected cells increased, whereas treated with Sp1 shRNA-NC, the cell viability of H9c2 cells were decreased again (Figure 5B). Accordingly, immunofluorescence showed that the positive rate of Sp1 in control group was lower than that in other groups, while the positive rate of Sp1 in OGD/R group and OGD/R+Sp1 shRNA-NC group was significantly higher. Furthermore, the positive rate of Sp1 in OGD/R+Sp1 shRNA group and OGD/R+AG-14361 group was significantly lower than that in OGD/R group and OGD/R+Sp1 shRNA-NC group (Figure 5C). These three results all demonstrated that both Sp1 suppression and PARP1 inhibition can prevent cardiomyocytes from OGD/R. Additionally, the regulation between Sp1 and PARP1 was positive.

To further confirm the regulation between Sp1 and PARP1, we infected H9c2 cells with Sp1 shRNA to downregulate Sp1 and AD-PARP1 adenovirus (MOI = 25) for 72 h to upregulate PARP1. Western blot analysis showed that under OGD/R exposition, the expression of PARP1 and autophagy-related proteins increased. However, when transfected H9c2 cells with Sp1 shRNA, the expression of PARP1 and autophagy-related proteins decreased, whereas infecting H9c2 cells with AD-PARP1 adenovirus upregulated the expression of PARP1 and autophagy-related proteins again (Figure 5D). In line with this, we performed real-time PCR. Results showed that compared to the OGD/R group, the expression of PARP1 mRNA and autophagy-related protein mRNAs decreased when H9c2 cells were transfected with Sp1 shRNA, while infecting H9c2 cells with AD-PARP1 adenovirus increased the expression of PARP1 mRNA and autophagy-related protein mRNAs (Figure 5E). As is mentioned above, treating cardiomyocytes with AD-PARP1 adenovirus could reverse the myocardial protection effect of Sp1 suppression. Taken together, Sp1 suppression prevented cardiomyocytes from OGD/R through PARP1 inhibition.

### PARP1 Inhibition Protected Cardiomyocytes From MIRI Through Inhibition of Autophagy, Which Was Targeted by Sp1 Suppression

To confirm the role and mechanism of PARP1 in the development of MIRI, we established a MIRI animal model by 45 min of ischemia via ligating left anterior descending branch (LAD) of the SD rats, and then reperfused for 24 h. The model was considered successfully established when electrocardiogram showed that the ST-segment elevated during myocardial ischemia period and dropped at least 50% during reperfusion period. Furthermore, the expressions of PARP1 and Sp1 in MIRI tissues are both significantly higher than those in control group, which were examined by immunofluorescence (Supplementary Figure 2). Then, we transfected Sp1 shRNA, negative control Sp1 shRNA-NC and AD-PARP1 adenovirus (MOI = 25) all for 72 h in SD rats, and used 10 μM AG-14361 for 1 h and 0.25 μM BEZ235 for 24 h to confirm the results from *in vitro* experiments.

**FIGURE 6:**
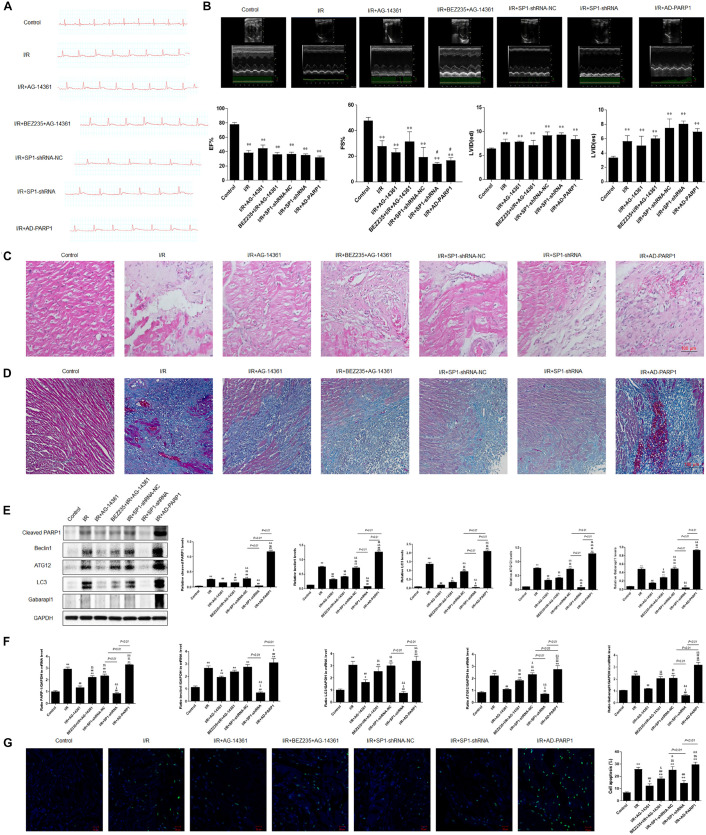
PARP1 inhibition protected cardiomyocytes from MIRI *in vivo* via inhibition of autophagy, which is targeted by Sp1 suppression. An MIRI rat model was established by 45 min of ischemia via ligating left anterior descending branch (LAD), and then refused for 24 h. MIRI-modeled SD rats were treated with Sp1 shRNA-NC, Sp1 shRNA, PARP1 inhibitor AG-14361 and AD-PARP1 adenovirus. **(A)** Electrocardiogram showed the ST-segment variation of SD rats when treated with Sp1 shRNA-NC, Sp1 shRNA, PARP1 inhibitor AG-14361 and AD-PARP1 adenovirus under MRI model. **(B)** Echocardiography showed the different left ventricular ejection fraction (LVEF%) and fractional shortening (FS%) of SD rats when treated with Sp1 shRNA-NC, Sp1 shRNA, PARP1 inhibitor AG-14361 and AD-PARP1 adenovirus under MIRI model. **(C,D)** Hematoxylin and eosin (H&E) and Masson trichrome staining were performed to assess myocardial fibrosis of SD rats when treated with Sp1 shRNA-NC, Sp1 shRNA, PARP1 inhibitor AG-14361 and AD-PARP1 adenovirus under MIRI model. Magnification: 100×. **(E)** Western blot analysis showed the expression of PARP1 and autophagy-related proteins of SD rats when treated with Sp1 shRNA-NC, Sp1 shRNA, PARP1 inhibitor AG-14361 and AD-PARP1 adenovirus under MIRI model. **(F)** Real-time PCR analysis showed the mRNA expression of PARP1 and autophagy-related proteins of SD rats when treated with Sp1 shRNA-NC, Sp1 shRNA, PARP1 inhibitor AG-14361 and AD-PARP1 adenovirus under MIRI model. **(G)** TUNEL assay showed the different apoptotic rate of SD rats when treated with Sp1 shRNA-NC, Sp1 shRNA, PARP1 inhibitor AG-14361 and AD-PARP1 adenovirus under MIRI model. Magnification: 100×. Compared with control group, *P < 0.05, **P < 0.01; compared with OGD/R group, ^#^*P* < 0.05, ^##^*P* < 0.01; compared with OGD/R+AG-14361 group, ^$^*P* < 0.05, ^$$^*P* < 0.01; compared with BEZ235+OGD/R+AG-14361 group, ^&^*P* < 0.05, ^&&^*P* < 0.01. *N* = 4 for each group.

Electrocardiogram showed that the ST-segment elevated when exposed to MIRI, compared to control group. The elevated ST-segment dropped after treating rats with AG-14361, whereas using BEZ235 elevated the ST-segment again. Moreover, compared to Sp1 shRNA-NC group, electrocardiogram showed that the ST-segment dropped in rats transfected with Sp1 shRNA, whereas infecting rats with AD-PARP1 adenovirus elevated the ST-segment again (Figure 6A). Consistently, echocardiography result showed that compared to control group, left ventricular ejection fraction (LVEF%) and fractional shortening (FS%) significantly decreased when exposed to MIRI. After treating rats with AG-14361, LVEF% and FS% increased, whereas using BEZ235 deteriorated LVEF% and FS% again. Furthermore, echocardiography revealed that LVEF% and FS% was increased in rats transfected with Sp1 shRNA, whereas infecting rats with Sp1 shRNA and AD-PARP1 adenovirus deteriorated FS%, compared to Sp1 shRNA-NC group (Figure 6B). Sp1 shRNA and AD-PARP1 showed less effect on LVEF% compared to Sp1 shRNA-NC group. Then, H&E and Masson trichrome staining were performed to assess myocardial fibrosis after MIRI. Compared with control group, the expression of fibrosis increased when exposed to MIRI. When rats were treated with AG-14361 or infecting rats with Sp1 shRNA, myocardial fibrosis was attenuated. However, using BEZ235 or infecting rats with Sp1 shRNA and AD-PARP1 adenovirus aggravated myocardial fibrosis (Figures 6C,D). According to western blot results, the expression of PARP1 and autophagy-related proteins decreased when rats were treated with AG-14361 or transfected with Sp1 shRNA, whereas rats were treated with BEZ235 or infected with Sp1 shRNA and AD-PARP1 adenovirus increased the expression of PARP1 and autophagy-related proteins again (Figure 6E). Consistently, real-time PCR showed that the expression of PARP1 and autophagy-related mRNAs decreased when rats were treated with AG-14361 or Sp1 shRNA, whereas BEZ235 or Sp1 shRNA and AD-PARP1 adenovirus increased the expression of PARP1 and autophagy-related mRNAs again (Figure 6F). Furthermore, TUNEL assay showed that the apoptotic rate decreased when rats were treated with AG-14361 or Sp1 shRNA, whereas BEZ235 or Sp1 shRNA and AD-PARP1 adenovirus increased the apoptotic rate (Figure 6G).

Taken together, these results demonstrated that PARP1 inhibition protects cardiomyocytes from myocardial ischemia-reperfusion injury through inhibition of autophagy by Sp1 suppression.

## Discussion

Myocardial ischemia-reperfusion injury (MIRI) has been reported to be associated with autophagy, and this disease can increase the level of autophagy in cardiomyocytes (Kloner and Jennings, 2001; Rochitte et al., 1998; Baines, 2011; Thapalia et al., 2014). It has been reported that autophagy, mitochondrial damage and endoplasmic reticulum stress (ERs) are important mechanisms of ischemia-reperfusion injury. Previous studies suggest that moderate autophagy can protect myocardium, while excessive autophagy can promote apoptosis and damage myocardial function (Thapalia et al., 2014). However, how to alleviate MIRI through targeting the pathway of autophagy remains elusive. In this work, we provide original findings on the mechanism underlying MIRI and autophagy. In our experiments, a cell model of oxygen-glucose deprivation/reperfusion (OGD/R) was adopted to simulate MIRI *in vitro*. We confirmed that PARP1 was activated by MIRI-induced myocardial autophagy. Treating cardiomyocytes with PARP1 inhibitor AG-14361, we observed that cardiac injury triggered by OGD/R was alleviated, whereas treating cardiomyocytes with NVP-BEZ235, a novel autophagy promoter, could reverse the myocardial protection effect that was mediated by PARP1 inhibition. Thus, PARP1 inhibition protected cardiomyocytes from OGD/R through inhibition of autophagy. Further exploration of the mechanism revealed Sp1 as a transcription factor of PARP1, which regulated the target gene of PARP1 through binding to its promoter during transcription. Additionally, silencing Sp1 prevented cardiomyocytes from OGD/R. Furthermore, we demonstrated that it was via PARP1 inhibition that Sp1 suppression prevented cardiomyocytes from OGD/R. *In vivo*, we established an MIRI animal model, and the same role and mechanism of PARP1 in the progression of MIRI have been verified. These results justified that Sp1 targeted PARP1 inhibition protected cardiomyocytes from MIRI via downregulation of autophagy. Therefore, PARP1 may be a therapeutic target for MIRI in the future.

Recent studies have proved that PARP1 inhibition can protect diabetic heart via activating SIRT1-PGC1 alpha axis (Waldman et al., 2018); Through activating SIRT1-induced inhibition of PARP1, post myocardial infarction infammation and cardiac remodeling can be prevented (Eid et al., 2020). Apart from cardiovascular diseases, deficiency of PARP1 can also alleviate pulmonary fibrosis (Zhang et al., 2018), while PARP1 overactivation can lead to hepatic fibrosis (Mukhopadhyay et al., 2014). Their results indicated that PARP1 inhibition can be a therapeutic target for the treatment of cardiovascular diseases. In our study, we are dedicated to exploring the effect of PARP1 inhibition in MIRI, which has rarely been studied before. OGD/R model was adopted to simulate MIRI *in vitro*. Our results showed that PARP1 was activated in OGD/R model (Figure 1). Moreover, it was demonstrated that PARP1 was inhibited by AG-14361, and PARP1 inhibition could protect cardiomyocytes from OGD/R (Figures 2A–C).

Autophagy begins in the early stage of myocardial ischemia and persists or even exacerbates in the late stage of reperfusion (Thapalia et al., 2014). Furthermore, autophagy is not only a pathophysiological process during MIRI, but also a potential regulated target to affect the progression of MIRI. Wang et al. reported that via regulating autophagy, PARP1 inhibition can attenuate cardiac fibrosis which is induced by myocardial infarction (Wang C. et al., 2018). Huang et al. indicated that DNA damage response 1 overexpression protected against the development of post-MI heart failure by enhancing autophagy and reducing apoptosis via the mTOR signaling pathway (Huang et al., 2019). In our study, autophagy was successfully induced by OGD/R model (Figures 2A–C). Then, a series of rescue experiments were conducted to prove that through downregulation of autophagy, PARP1 inhibition can protect cardiomyocytes from OGD/R. AG-14361 was used to suppress the expression of PARP1, while BEZ235 was used to increase the effect of autophagy related cardiac injury. We discovered that PARP1 inhibition could alleviate the cardiac damage of OGD/R, while increasing the extent of autophagy could reverse the myocardial protection effect of PARP1 inhibition (Figures 2D–G). Therefore, PARP1 inhibition can protect cardiomyocytes from OGD/R through inhibition of autophagy.

It has been reported that Sp1 is a transcription factor of the Sp/Kruppel-like factor family, and it plays an important role in apoptosis, differentiation and cell growth (Vizcaíno et al., 2015). Furthermore, Sp1 can regulate the gene expression via activating the transcription of many cellular genes which contain Sp1 binding sites in their promoters (Safe et al., 2014). Our research showed that Sp1 was selected as a transcription factor of PARP1, and there was a targeted regulation between Sp1 and PARP1. Here, we also verified that Sp1 could regulate the target gene of PARP1 through binding to its target gene promoter during transcription (Figures 3D,E). Wei et al. argued that downregulate Sp1 could decrease the progression of carcinogenesis in pancreatic cancer (Wei et al., 2004). Additionally, previous researches have demonstrated that Sp1 is a drug target. A number of antineoplastic agents inhibit the expression of Sp1 (Safe et al., 2014), which are effective on tumors. Therefore, we speculate that Sp1 suppression might have myocardial protective effect in MIRI. We transfected H9c2 cells with Sp1 shRNA to inhibit Sp1 and infected H9c2 cells with AD-PARP1 adenovirus to upregulate PARP1. Sp1 suppression attenuated cell autophagy and alleviated cell apoptosis, showing a myocardial protective effect (Figures 4B,D). Suppressing Sp1 can lead to PARP1 inhibition, which induces autophagy inhibition, and finally results in cardioprotection from MIRI (Figure 5).

Consistently, we also observed the same myocardial protective effect *in vivo* by inhibiting Sp1. Furthermore, Sp1 suppression could also protect the cardiac function of MIRI rats and palliate their cardiac remodeling.

There are also limits in our research such as LC3 immunofluorescence only represented the autophagosome formation, not the autophagy flux, and mRFP-GFP-LC3 label should be used in the experiment. Furthermore, H9c2 cells were used in all experiments *in vitro*, and the cultured neonatal rat cardiomyocytes were not involved in the present study. *In vivo*, the experiments of detecting quantification of heart infarct size and fibrosis were not performed. Further investigations should aim at those unsettled obstacles, and these will be a part of our future research.

In conclusion, we have discovered the role and mechanism of PARP1 in the progression of MIRI. For the first time, we demonstrated that PARP1 inhibition protected cardiomyocytes from MIRI through inhibition of autophagy, which was targeted by Sp1 suppression. This exposits a promising therapeutic target in treating myocardial reperfusion injury in the future.

## Data Availability Statement

The original contributions presented in the study are included in the article/Supplementary Material, further inquiries can be directed to the corresponding author/s.

## Ethics Statement

The animal study was reviewed and approved by Shanghai General Hospital Affiliated to Shanghai Jiao Tong University [Permission Number: SYXK (Su) 2017-0007].

## Author Contributions

YX and HL designed the experiments. YX and BW conducted the experiments and wrote the manuscript. XL, YD, YL, YZ, and FZ amended the protocol of the experiments. All authors revised and approved the manuscript.

## Conflict of Interest

The authors declare that the research was conducted in the absence of any commercial or financial relationships that could be construed as a potential conflict of interest.

## Footnotes

1 https://bigd.big.ac.cn/databasecommons/

## Supplementary Material

According to OGD real-time PCR analysis, the mRNA expression of autophagy related genes increased to the peak at 6 h and then decreased, compared to control group (Supplementary Figure 1A). Therefore, 6 h was chosen as the model OGD time. Identically, OGD/R real-time PCR analysis confirmed that the mRNA expression of autophagy-related genes increased to the peak at 2 h and then decreased, compared to control group (Supplementary Figure 1B). Therefore, 2 h of reoxygenation was chosen as the model time.

The expression of Sp1 and PARP1 were both confirmed in sham control and MIRI tissues by immunofluorescence. Our results showed that the expressions of PARP1 and Sp1 in MIRI tissues are both significantly higher than those in control group (Supplementary Figure 2).

A model depicting the mechanism of Sp1 targeted PARP1 inhibition protects cardiomyocytes from myocardial ischemia–reperfusion injury via downregulation of autophagy was added as a Graphical Abstract.

Supplementary Figure 1 H9c2 cells were cultured in an oxygen/glucose-deprived incubator for 6 h, and then reperfused by oxygen and glucose for 2 h to cultivate an OGD/R model. **(A)** Real-time PCR showed that when OGD was 6 h, the mRNA expression of each autophagy related genes was the highest. **(B)** Real-time PCR showed that when OGD/R was 2 h, the mRNA expression of each autophagy related genes was the highest. Compared with control group, ^∗^*P* < 0.05, ^∗∗^*P* < 0.01. *N* = 4 for each group.

Supplementary Figure 2 Immunofluorescence examined the expression of Sp1 and PARP1 in sham control and MIRI tissues. **(A)** The expression of PARP1 was significantly higher in MIRI tissues than that in sham control group. **(B)** Compared to sham control group, the expression of Sp1 expression was conspicuously increased in MIRI tissues. Magnification: 200×. *N* = 4 for each group.

Supplementary Graphical Abstract A model depicting the mechanism of Sp1 targeted PARP1 inhibition protects cardiomyocytes from myocardial ischemia–reperfusion injury via downregulation of autophagy.

## Abbreviations

AD: adenovirus
AMI: acute myocardial infarction
BEZ235: NVP-BEZ235
ChIP: chromatin immunoprecipitation
DAPI-4,6: diamidino-2-phenylindole
ECG: electrocardiogram
FS: fractional shortening
H&E staining: hematoxylin and eosin staining
LAD: left anterior descending coronary artery
LC3: micro-tubule-associated protein1 light chain 3
LVEF: left ventricular ejection fraction
MIRI: myocardial ischemic-reperfusion injury
NC: negative control
OGD/R: oxygen-glucose deprivation/reperfusion
PARP1: poly(ADP-ribose) polymerase-1
PCI: percutaneous coronary intervention
Real-time PCR: real-time polymerase chain reaction
ROS: reactive oxygen species
Sp1: specificity protein 1
TF: transcription factor
TUNEL: terminal dexynucleotidyl transferase (TdT)-mediated dUTP nick end labeling
3′ UTR: 3′ untranslated regions.
