# Supplementary figures and images for: Sp1 Targeted PARP1 Inhibition Protects Cardiomyocytes From Myocardial Ischemia–Reperfusion Injury via Downregulation of Autophagy
Source: Front Cell Dev Biol. 2021 May 25;9:621906. doi: 10.3389/fcell.2021.621906 (PMC8190009; doi:10.3389/fcell.2021.621906)

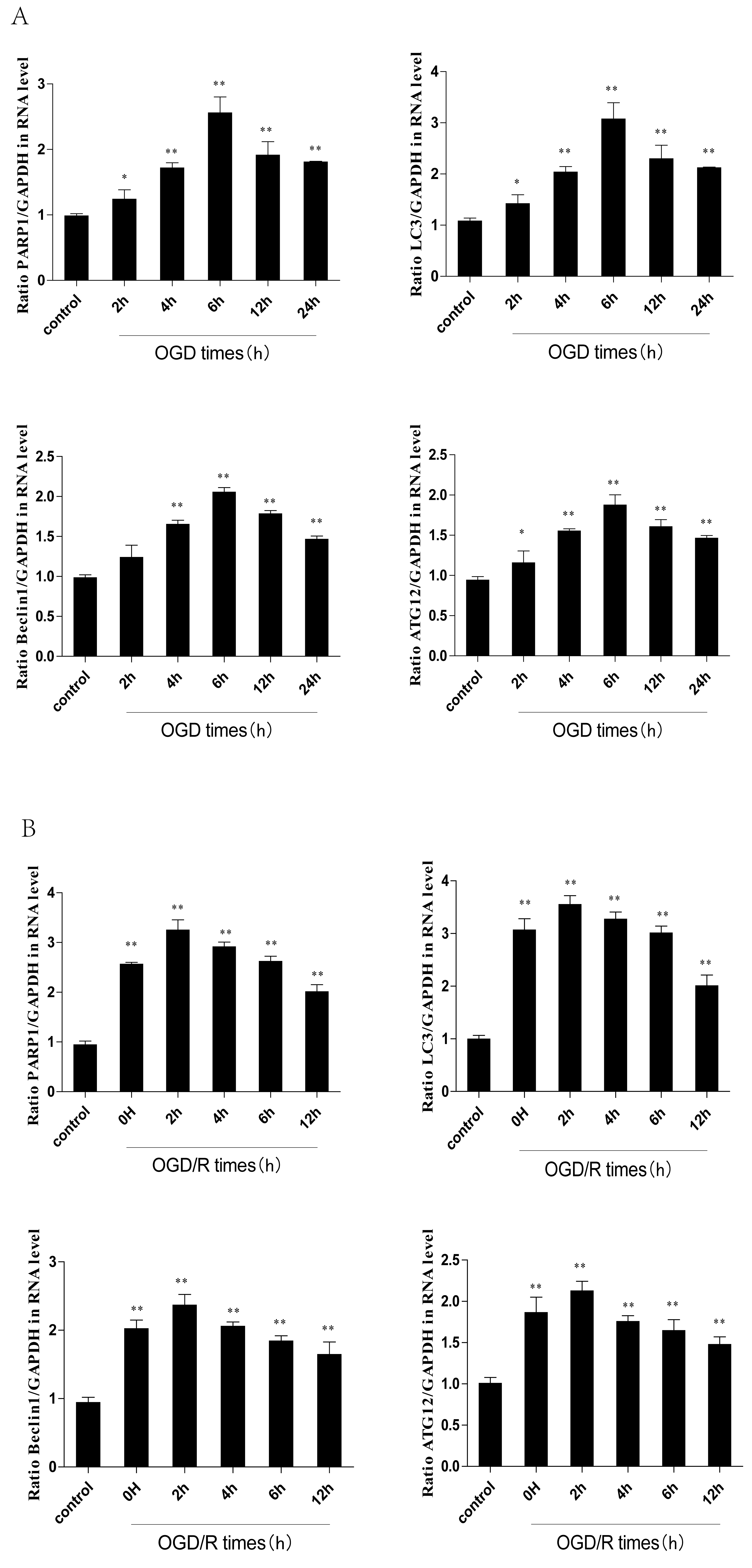

Supplement: Supplementary Figure 1 — H9c2 cells were cultured in an oxygen/glucose-deprived incubator for 6 h, and then reperfused by oxygen and glucose for 2 h to cultivate an OGD/R model. (A) Real-time PCR showed that when OGD was 6 h, the mRNA expression of each autophagy related genes was the highest. (B) Real-time PCR showed that when OGD/R was 2 h, the mRNA expression of each autophagy related genes was the highest. Compared with control group, ∗P < 0.05, ∗∗P < 0.01. N = 4 for each group. [file Image_1.TIF]

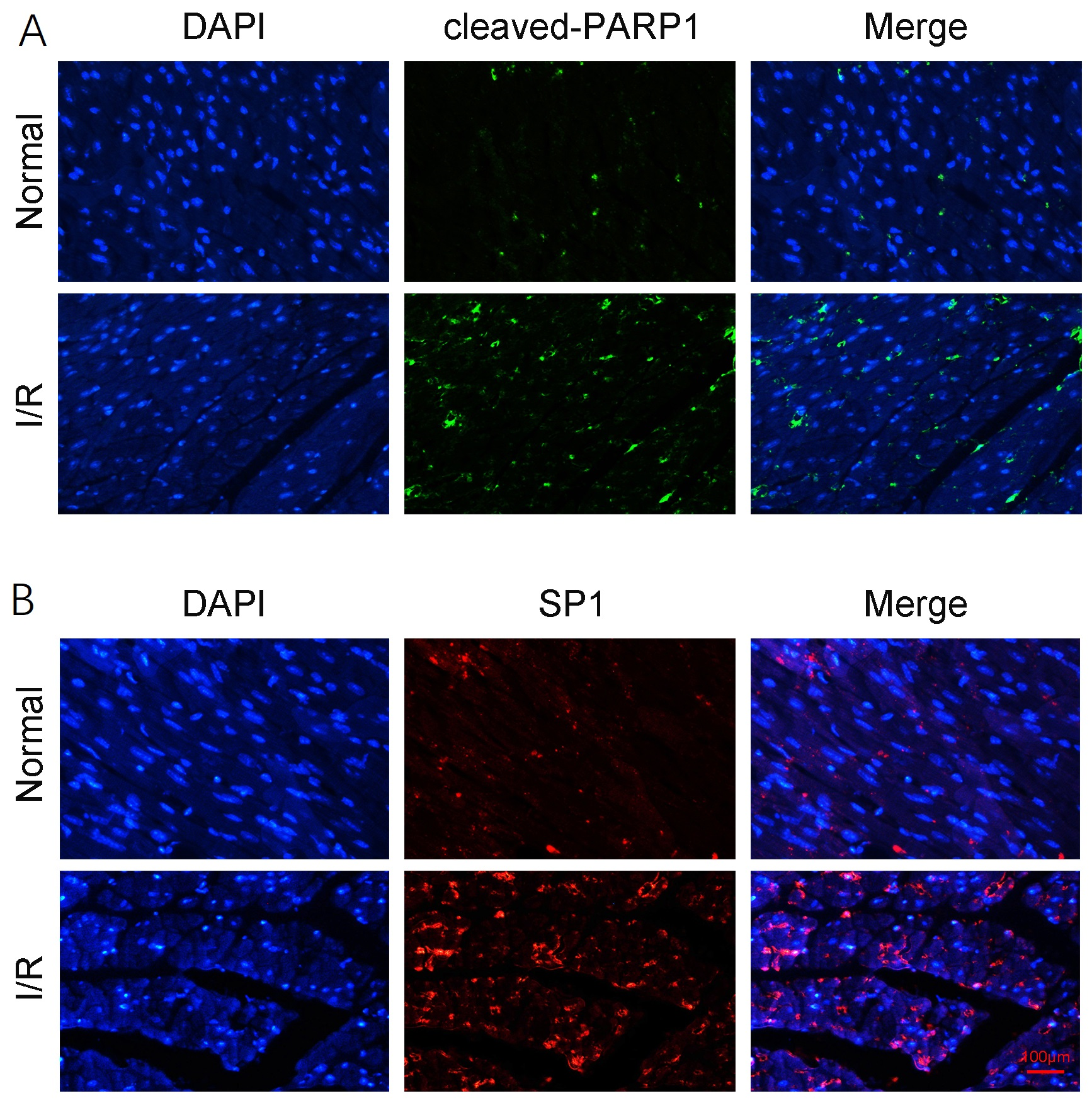

Supplement: Supplementary Figure 2 — Immunofluorescence examined the expression of Sp1 and PARP1 in sham control and MIRI tissues. (A) The expression of PARP1 was significantly higher in MIRI tissues than that in sham control group. (B) Compared to sham control group, the expression of Sp1 expression was conspicuously increased in MIRI tissues. Magnification: 200×. N = 4 for each group. [file Image_2.TIF]

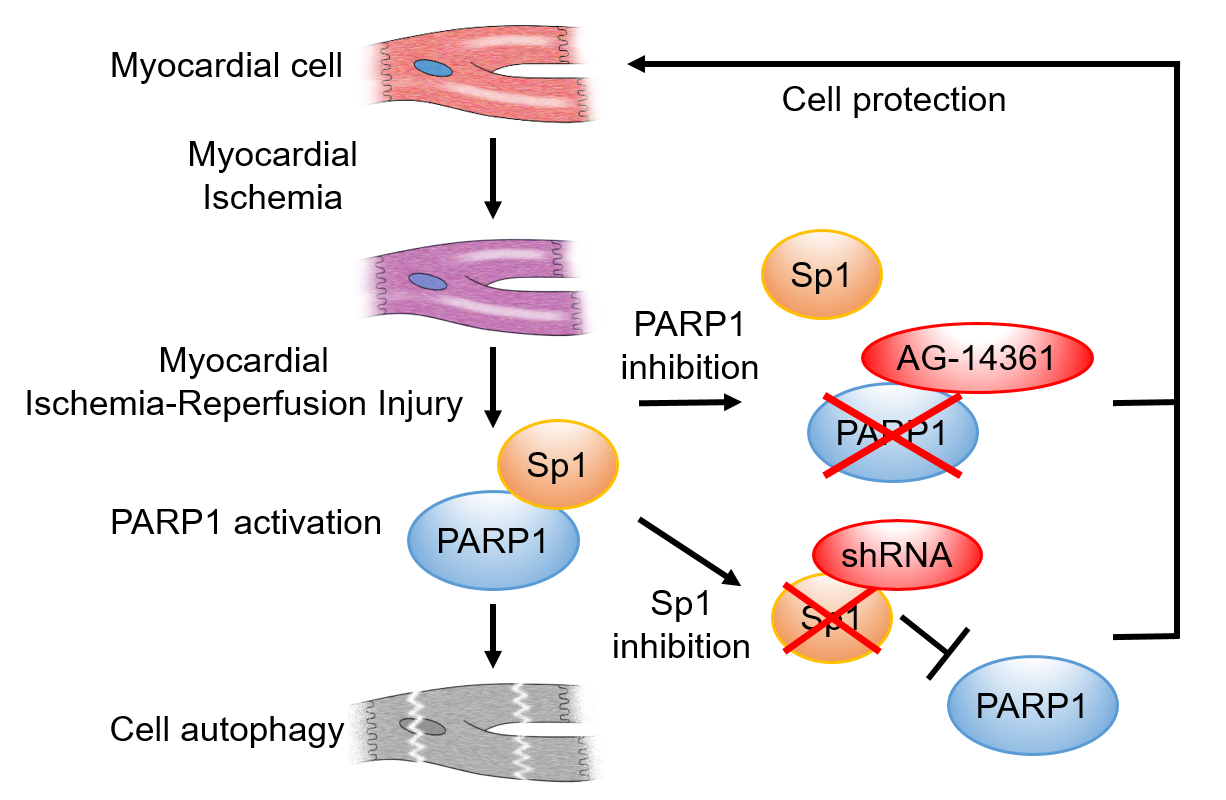

Supplement: Supplementary Graphical Abstract — A model depicting the mechanism of Sp1 targeted PARP1 inhibition protects cardiomyocytes from myocardial ischemia–reperfusion injury via downregulation of autophagy. [file Image_3.TIF]
